# Does Caffeine Affect Dental Implant Stability? A Prospective Cohort Study

**DOI:** 10.30476/DENTJODS.2021.87624.1274

**Published:** 2022-06

**Authors:** Mohammad Jafarian, Reza Tabrizi, Sajjad Haghi, Shervin Shafiei

**Affiliations:** 1 Dept. of Oral and Maxillofacial Surgery, School of Dentistry, Shahid Beheshti University of Medical Sciences, Tehran, Iran; 2 Dental Student, Shahid Beheshti University of Medical Sciences, Tehran, Iran; 3 Postgraduate Student, Dept. of Oral and Maxillofacial Surgery, School of Dentistry, Shahid Beheshti University of Medical Sciences, Tehran, Iran

**Keywords:** Dental implants, Caffeine, Maxilla, Osseointegration

## Abstract

**Statement of the Problem:**

Caffeine intake affects bone metabolism through inhibition of osteoblast proliferation.

**Purpose:**

This study aims to assess the effect of caffeine consumption on implant stability in the healing period of patients.

**Materials and Method:**

A prospective cohort study is designed to assess implant stability in the posterior of the maxilla. Patients were divided into two groups based on daily caffeine intake as group 1 with consumption of 400 mg/daily caffeine or more, and group 2 with consumption of 100 mg/daily caffeine or less. The implant stability was measured by resonance frequency analysis (RFA). The mean implant stability quotients (ISQs) were calculated. RFA measurements were made at 4, 6 and 8 weeks after implant placement.

**Results:**

A total of 102 patients were studied (51 in each group). The mean of ISQ was 43.49± 2.32 in the group 1 and 42.78±2.34 in the group 2 at four weeks after insertion. The mean of ISQ was 50.86±3.06 in group 1 and 51.37±2.44 in the group at six weeks after implant placement. At eight weeks after implant surgery, the mean of ISQ was 56.78±3.77 in the group 1 and 57.84±1.82 in the group 2. The mean of ISQ between the two groups at 4, 6 and 8 weeks after implant placement was not statistically different. (*p*= 0.13, *p*= 0.36 and *p*= 0.08 respectively) The repeated measure test indicated a similar increase in ISQ in various study times in the two studied groups (*p*=0.47, df=1, F=0.52).

**Conclusion:**

Acquired data suggest that caffeine intake may not have a negative effect on implant stability in the healing period at the posterior of the maxilla.

## Introduction

Caffeine is a well-known substance, which is found in coffee, tea, energy drinks, chocolate, and so on. Up to 80% of people consume caffeine around the world [ 1 ]. It is believed that caffeine could inhibit osteoblast proliferation *in vitro* by the increase of cyclic adenine monophosphate (cAMP) and the inhibition of the intracellular phosphodiesterase [ 2 ]. In an animal study, the use of caffeine resulted in lower volume of bone, reduced bone mineral density and delayed bone repair [ 3 ]. It was reported that the consumption of caffeine has increased the orthodontic tooth movement [ 4 ].

 Implant stability is defined as the absence of clinical mobility, which is crucial for osseointegration. Resonance frequency analysis (RFA) stability measurement applies a bending load, which is similar to the clinical load and indicates the stiffness of the implant-bone connection [ 15 ]. To the best of our knowledge, no study has been done to assess the effect of caffeine intake on the stability and healing of dental implants.

The purpose of this study is to address the following question: Do patients consuming caffeine have lower implant stability during the healing period? We hypothesized that caffeine affects the bone healing process and decreases implant stability. Therefore, the aim of this study was to compare implant stability in patients with and without caffeine consumption.

## Materials and Method

The authors designed a prospective cohort study. The sample was derived from patients who attended the Oral and Maxillofacial Department of Shahid Beheshti University of Medical Sciences and a private clinic between September 1, 2018, and December 31, 2019. The Medical Ethics Committee of Shahid Beheshti University of Medical Sciences has approved the study (IR.SBMU. DRC.REC.1397.60). Patients eligible for study inclusion had a partially edentulous area at the posterior of the maxilla and received a dental implant for restoration there. The exclusion criteria were defined as any systemic disease affecting bone metabolism, smoking habit, need for augmentation and or sinus lift and those who refused participation or failed to return for follow up.

Patients were divided up into two groups as group 1, in which patients took 400 mg/daily or more of caffeine, and group 2, in which the participants took caffeine for 100 mg/ daily or less. The estimated amount of caffeine intake is shown in Table 1. 

**Table 1:** The amount of caffeine in various beverages

Coffee drinks	Size in oz(ml)	Caffeine (mg)
Brewed	8(237)	96
Espresso	1 (30)	64
Instant	8(237)	62
Brewed black tea	8 (237)	47
Cola	8(237)	22
Energy drink	8(237)	29
Energy Shot	1 (30)	215
Green tea	8 (237)	25

### Implant Surgery

All implants were placed in healed bone at least 12 weeks after tooth removal. A crestal incision was made on the alveolar ridge with two short releasing with preserving gingival tissue in the proximal and distal aspects adjacent to neighbor teeth. Instrumentation was performed based on the company guidelines. A dental implant (SGS, Switzerland) with 4.5mm x 10mm size was placed at the first or second molar area in the posterior of the maxilla. A smart peg was connected to the fixture.

### Implant stability measurements

Two examiners who were blinded to the groups evaluated the implant stability. The stability of the implants was measured by RFA. An Osstell device (Osstell, Gothenburg, Sweden) was used. 

The buccolingual and mesiodistal directions were measured. Next, the mean implant stability quotients (ISQs) were calculated. RFA measurements were made at 4, 6 and 8 weeks after implant placement.

### Statistical Analysis

The statistical analysis was performed using statistical package for the social sciences (SPSS) version 21 software (SPSS Inc., IBM, USA). The repeated measurement test was used to compare ISQ values between the two groups at each measurement time point. An Independent T-test was used to compare the mean of age between the studied groups. A p value of <0.05 was considered statistically significant. An inter-examiner reliability analysis (Kappa test) was applied to determine the agreement between the two examiners.

## Results

A total of 102 patients who had an implant at the posterior of the maxilla were divided into two groups (51 patients in each group). Group 1 consisted of 30 males and 21 females, and group 2 included 29 males and 22 females. The gender distribution between the two groups was not statistically different (*p*= 0.50). The mean age was 38.27±10.07 years in group 1 and 40.24± 7.67 years in the group 2. There was no difference in the mean of age between the two groups (*p*= 0.27) (Table 2).

**Table 2:** Comparison of variables between the two groups

Variables	Group1	Group2	*p* Value
Age (years)	38.27±10.07	40.24±7.67	*p*= 0.27*
Gender	30 males, 21 females	29 males, 22 females	*p*= 0.50**

*Independent T-test **chi-square test

**Figure 1:**
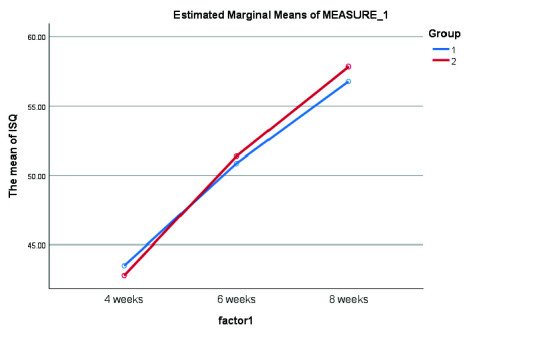
The mean of implant stability quotients (ISQ) at 4, 6, 8 weeks after implant placement in two groups

The mean of ISQ was 43.49±2.32 in the group 1 and 42.78±2.34 in the group 2 at four weeks after insertion. The mean of ISQ was 50.86±3.06 in the group 1 and 51.37±2.44 in the group at six weeks after implant placement. Eight weeks after surgery, the mean of ISQ was measured to be 56.78±3.77 in the group 1 and 57.84±1.82 in the group 2. Analysis of the data did not demonstrate any difference for the mean of ISQ between the two groups at 4, 6 and 8 weeks after implant placement (*p*= 0.13, *p*= 0.36 and *p*= 0.08, respectively) (Table 3). The repeated measure test indicated a similar increase in ISQ in various study times in the two studied groups (*p*= 0.47, df=1, F=0.52) (Figure 1). The inter- examiner reliability for the examiners was found to be Kappa=0.90 (*p*< .0.001), 95% CI, which shows almost a perfect agreement between the examiners.

**Table 3:** Comparison of implant stability quotients (ISQ) in study times between two groups

Outcomes	Group 1	Group 2	Independent T-test
The mean of ISQ at four weeks after the implant placement	43.49±2.32	42.78±2.34	*p*= 0.13
The mean of ISQ at six weeks after the implant placement	50.86±3.06	51.37±2.44	*p*= 0.36
The mean of ISQ at eight weeks after the implant placement	56.78±3.77	57.84±1.82	*p*= 0.08

## Discussion

Caffeine (1,3,7-trimethyl xanthine) is the most commonly consumed psychoactive agent around the world. The possible effect of caffeine on bone metabolism was evaluated in a series of animal and clinical studies [ 6 - 8 ]. It is believed that caffeine has a negative effect on bone metabolism [ 9 ]. In this study, we studied the effect of caffeine with a dose of 400 mg/daily on implant stability during the healing period.

The dose of caffeine was estimated according to patients’ self-reports. Various beverages with caffeine content were consumed which were documented. ISQ measurements at 4, 6, 8 weeks after implant placement indicated no significant difference in the mean of ISQ in patients who consumed more than 400 mg/daily and less than 100 mg/daily. It was suggested that the dose of caffeine more than 400 mg is toxic for human kind and less than 100 mg does not have a clinical effect on bone [ 10 ]. It was suggested that more than 300 mg daily caffeine (approximately 514 g, or 18 oz, brewed coffee) increases bone loss in human [ 11 ]. It could be interpreted that the use of caffeine in the healing period of dental implants does not have a negative effect on implant stability. Generally, we expect a low stability in D4 bone at the posterior of the maxilla. Therefore, any detrimental effect of caffeine could be easily observed. In our review, we did not find any similar study, which evaluated the effect of caffeine on implant stability during the healing period. 

Duarte *et al*. [ 12 ] studied the effect of caffeine on the early stage of bone healing and also bone density in rats. They concluded that a high daily caffeine intake may have a negative effect on the early stages of bone healing, but does not change bone density 56 days after administration [ 12 ]. A hypothesis for the possible role of caffeine in bone metabolism is its effect on calcium metabolism and the proliferation of osteoblast-like cells [ 12 ]. Caffeine raises urinary calcium excretion by a decrease renal reabsorption and calcium absorption, which leads to a negative calcium balance [ 3 , 13 - 14 ]. There were many studies, which have reported the effect of caffeine on osteoblast function *in vivo* [ 11 , 13 , 19 ]. 

Tasuang *et al*. [ 13 ] studied the effect of caffeine on osteoblasts derived in newborn Wistar rats’ calvaria. They reported that caffeine had possible deleterious effect on the osteoblasts viability, which may increase the rate of osteoblasts apoptosis [ 13 ]. Rapuri *et al*. [ 11 ] reported that caffeine could stimulate 1,25(OH)2D3 stimulated vitamin D receptor protein expression, which reduces human osteoblast cells through 1,25(OH)2D3 mediated actions. Bezerra *et al*. [ 17 ] studied the effects of caffeine on ligature-induced bone loss, trabecular bone area and post-extraction bone healing in rats. They found that caffeine consumption resulted in bone loss in ligated teeth and delayed bone healing in post-extraction sockets. Sakamoto *et al*. [ 14 ] indicated that caffeine did not increase bone loss in rats. Ferreira *et al*. [ 18 ] studied the effect of caffeine and/or estrogen deficiency on trabecular bone area and healing. They concluded than caffeine would influence the bone healing, while estrogen deficiency disturbs trabecular bone area mainly.

In dental research, caffeine was studied in orthodontic tooth movements. The study showed that drinking coffee might accelerate tooth movement in orthodontic treatment [ 4 ] Shirazi *et al*. [ 19 ], studied caffeine intake in rats and orthodontic tooth movement. They demonstrated that caffeine intake resulted in decreased root resorption and consumption with concentrations of 2 g/L and 3 g/L inhibited orthodontic tooth movement due to its influence on osteoclast numbers [ 19 ]. Moreover, it was shown that caffeine caused a delay in bone healing in socket following tooth extraction in rats [ 19 ].

Concerning the limitation of the present study, the estimation of the amount of caffeine intake by patients was performed based on self-reports, which may not be very precise. 

## Conclusion

Our findings indicate that caffeine intake may not have a negative effect on implant stability in the healing period at the posterior of the maxilla.

## Conflict of Interest

The authors declare that there is no conflict of interest.
